# A murine model of prurigo nodularis-like skin lesions induced by persistent scratching under type 2 inflammatory conditions

**DOI:** 10.3389/fimmu.2025.1648830

**Published:** 2025-10-28

**Authors:** Jiali Wu, Xiahong Li, Siqi Huang, Siyi Tang, Kaoyuan Zhang, Jiamin Yan, Xiaofan Chen, Xia Dou

**Affiliations:** ^1^ Department of Dermatology, Peking University Shenzhen Hospital, Institute of Dermatology, Shenzhen Peking University-The Hong Kong University of Science and Technology Medical Center, Shenzhen, China; ^2^ Shenzhen Key Laboratory for Translational Medicine of Dermatology, Institute of Dermatology, Shenzhen Peking University - the Hong Kong University of Science and Technology Medical Center, Shenzhen, Guangdong, China; ^3^ Biomedical Research Institute, Shenzhen Peking University - the Hong Kong University of Science and Technology Medical Center, Shenzhen, Guangdong, China

**Keywords:** prurigo nodularis, preclinical model, type 2 inflammation, Th17/Th22 skewing, dermal fibrosis

## Abstract

**Introduction:**

Prurigo nodularis (PN) is a chronic inflammatory dermatosis characterized by robust pruritus and highly keratinizing nodules sympathetically distributed on the trunk and extensor sides of the limbs with an impact on quality of life and socioeconomic burden. To investigate the pathogenesis and develop novel therapeutic approaches, a preclinical animal model recapitulating the PN phenotypes is needed.

**Methods:**

We constructed a novel PN-like mouse model by applying repetitive mechanical scratching in the context of type 2 inflammation. We utilized 6-8-week-old C57BL/6 mice for a 28 - day modeling study. The modeling protocol consisted of daily MC903 treatment plus 100 cell scraper scratches for the first 14 days, followed by alternate-day MC903 with continued daily scratching for the subsequent 14 days, with bandaging applied to prevent spontaneous scratching.

**Results:**

Histological analysis of lesions revealed aberrant epidermal hyperplasia and differentiation, dermal fibrosis, inflammatory infiltration, angiogenesis, increased intraepidermal nerve fiber density, and enhanced nerve fiber sprouting. Immunological characterization revealed a mixed Th1/Th2/Th17/Th22 profile with a skewing toward Th17/Th22 polarization.

**Conclusion:**

This optimized model faithfully recapitulates the key clinicopathological features of PN patients, providing a robust preclinical platform for investigating disease mechanisms and evaluating potential therapeutics.

## 1 Introduction

Prurigo nodularis (PN) is a chronic, intensely pruritic skin disorder characterized by symmetrically distributed hyperkeratotic nodules primarily affecting the trunk and extensor surfaces of the limbs (1). The disease predominantly affects African Americans and Asians and imposes a substantial burden on patients’ quality of life and socioeconomic well-being (2–4). Among chronic pruritic dermatoses, PN is associated with the greatest intensity and frequency of pruritus (5, 6). It often coexists with other inflammatory skin diseases, particularly atopic dermatitis (AD), a prototypical type 2 inflammatory disease, which is the most common cutaneous comorbidity observed in PN patients (7).

The characteristic nodular lesions of PN are thought to arise from repetitive mechanical trauma driven by persistent “itch-scratch” cycles in the context of neuroimmune dysregulation (8). Histopathologically, PN nodules exhibit pronounced hyperkeratosis, pseudoepitheliomatous hyperplasia, dermal fibrosis with thickened collagen, increased papillary capillaries, dense perivascular and interstitial inflammatory cell infiltration, and neural structural abnormalities (9–11). These changes are underpinned by a complex immune dysregulation involving Th1, Th2, Th17, and Th22 cells and their associated cytokine networks (7).

Although novel targeted therapies have emerged in recent years, their efficacy in resolving established PN nodules remains limited (8, 12–14). The lack of representative animal models that faithfully recapitulate the clinical and immunopathological features of PN has been a major obstacle to mechanistic studies and therapeutic development. Therefore, establishing a reliable PN-like mouse model is critical for advancing our understanding of the disease and preclinical evaluation of novel interventions.

In this study, we established a murine model with PN-like lesions by applying repetitive mechanical scratching under a type 2 - skewed inflammatory milieu. The resulting lesions closely resembled human PN nodules in histological alterations, inflammatory cell infiltration, and immune cell composition. This model offers a valuable experimental platform for elucidating the pathogenesis of PN and for exploring innovative therapeutic approaches.

## 2 Materials and methods

### 2.1 Mouse models

This model utilized C57BL/6 mice aged 6–8 weeks, with a modeling duration of 28 days. Topical MC903, known as calcipotriol, was widely used to induce AD-like inflammation (15). During the first 14 days, the shaved dorsal skin of mice was treated daily with 2 nmol MC903 diluted in 20 μL ethanol or 20 μL ethanol alone. After the skin was slightly dry, mechanical stimulation mimicking PN was induced by scratching the skin 100 times with a cell scraper. In the subsequent 14 days, 20 μL ethanol or 20 μL 2 nmol MC903 was applied every other day, while the mice scratched their backs 100 times each day. Throughout the experimental period, the shaved dorsal skin (approximately 2 cm × 3.5 cm area) of mice was subjected to daily standardized mechanical stimulation: an experienced technician used a 1.7 cm-wide cell scraper to scratch the defined area, with one complete back-and-forth motion counted as a single scratch, repeated 100 times daily at an intensity carefully controlled to reach just below the threshold of causing bleeding. Following each scratching session, the damaged skin was immediately wrapped with a bandage to prevent additional scratching by the mice.

On day 29, mice were humanely euthanized for harvesting and analysis of back skin. Four experimental groups were established: (1) EtOH group: ethanol application without scratching throughout the modeling period; (2) scratching (SC) group: daily ethanol application with daily scratching for the first 14 days, followed by ethanol application every other day with daily scratching for the remaining 14 days; (3) AD-like group: MC903 application without scratching throughout the modeling period; (4) PN-like group: daily MC903 application with daily scratching for the first 14 days, followed by MC903 application every other day with daily scratching for the remaining 14 days.

### 2.2 Histopathological assessment of skin

Skin specimens were fixed in 10% formalin (Phygene, Fuzhou, China) and embedded in paraffin. Slides of 5-μm-thick sections were prepared and stained with hematoxylin and eosin (H&E). Masson’s trichrome (MT) staining was using Masson’s Trichrome Stain Kit (Solarbio, Beijing, China) according to the manufacturer’s instructions. The eosinophil infiltration was evaluated by Chromotropic acid 2R staining using the Eosinophil Staining Kit (Solarbio, Beijing, China), and mast cells (MCs) were stained by toluidine blue staining (Solarbio, Beijing, China). Slides were then digitally scanned by Aperio CS2 scanner (Leica, Germany) and analyzed using software QuPath (v0.5.1) (16). The measurements of epidermal and dermal thickness in H&E staining and positively stained cells and area were evaluated from at least six randomly selected fields of each sample using the software ImageJ.

### 2.3 Immunohistochemistry analysis

Following deparaffinization, antigen retrieval and blocking the nonspecific binding, slides were incubated with primary antibodies against Cytokeratin 5/K5 (ab64081), Cytokeratin 10/K10 (ab76318), Loricrin (ab85679), CD31 (ab28364), PGP9.5 (ab8189) obtained from Abcam (Cambridge, UK); primary antibody against F4/80 obtained from Cell Signaling Technology (70076S, Danvers, USA); primary antibody against Cytokeratin 16/K16 obtained from Proteintech (17265-1-AP, Chicago, USA). Biotinylated secondary antibodies for immunohistochemistry (IHC) staining were from Beyotime Biotechnology (A0208, Shanghai, China). The signaling detection of IHC slides was performed by the DAB kit (ZSGB-BIO, Beijing, China). Slides were then digitally scanned by Aperio CS2 scanner (Leica, Germany). The percentage of positively stained area (CD31 and F4/80) was calculated at least six fields per each section by the ImageJ software.

### 2.4 Intraepidermal nerve fiber density quantification

PGP9.5 positive nerve fibers were used for the following analysis. Only single intraepidermal nerve fibers (IENFs) that cross the papillary dermis-epidermal junction were considered while secondary branching or fragments were not considered (17). IENF density (IENFD) was counted by the the total number of IENFs per site divided by the total length of the epidermis (fibers/mm) (18). Each section was calculated by at least two examiners who were researchers by the QuPath (v0.5.1) software at three fields.

### 2.5 RNA extraction and quantitative real-time polymerase chain reaction

Total RNA was extracted from skin tissues using TRIzol (Invitrogen, USA) according to manual instruction, which was qualified and quantified using an Agilent 2100 Bioanalyzer (Agilent, CA, USA). cDNA was generated by PrimeScript™ RT kit (Takara, Beijing, China). The expression of transcripts was analyzed for various genes using the CFX96 (Bio-rad) thermocycler. The relative expression was calculated by the 2-ΔΔCT method and β-actin was used to normalize. All primers are shown in **Supplementary Table S1**.

### 2.6 RNA sequencing and data analysis

Library preparation is performed using Optimal Dual-mode mRNA Library Prep Kit (BGI-Shenzhen, China). Sequencing was performed on DNBSEQ platform with PE150. Clean data using the SOAPnuke (19) were obtained by raw data that filtered out low-quality reads, adapter contaminants, etc. Software HISAT (20) was used for reads alignment to a reference genome (GRCm39.v2201) and Bowtie2(v2.3.4.3) (21) was used to map gene sequence. Gene expression quantification was performed using RSEM (v1.3.1) (22). DESeq2 (v1.4.5) (23) was employed for differentially expressed genes (DEGs), with criteria set as false discovery rate (FDR) <0.05 and |log2 fold change| > 1.

### 2.7 Protein-protein interaction network analysis and identification of hub genes

The Protein-protein interaction (PPI) network was constructed with STRING database (https://cn.string-db.org/) (24) to predict the internal connections among the selected DEGs, with a confidence score threshold set to 0.4. Network visualization was performed using Cytoscape software (Version 3.10.4) (25). The CytoHubba plugin was used to rank the nodes in the target network based on Maximal Clique Centrality (MCC), and the top 15 genes were selected as hub genes.

### 2.8 Statistical analysis

Statistical analyses were performed by Graphpad Prism (v10.1.0) software. One-way ANOVA followed by Tukey’s multiple comparison test, or Kruskal-Wallis test followed by Dunn’s multiple comparison test was performed among multiple groups relying on whether the data were normally distributed. The gene sets defined for gene set variation analysis (GSVA) were derived from the website Metascape (26), with detailed gene sets provided in **Supplementary Table S2**. GSVA was conducted by “GSVA” R package with R (v4.4.2). The number of samples used in each experiment was indicated in the figure legends. * *P* < 0.05 was considered statistically significant. The significance levels of the data were defined as *P < 0.05, **P < 0.01, ***P < 0.001, and ****P < 0.0001.

## 3 Results

### 3.1 Significant thickening of epidermis and dermis in PN-like mouse model

**Figure 1:**
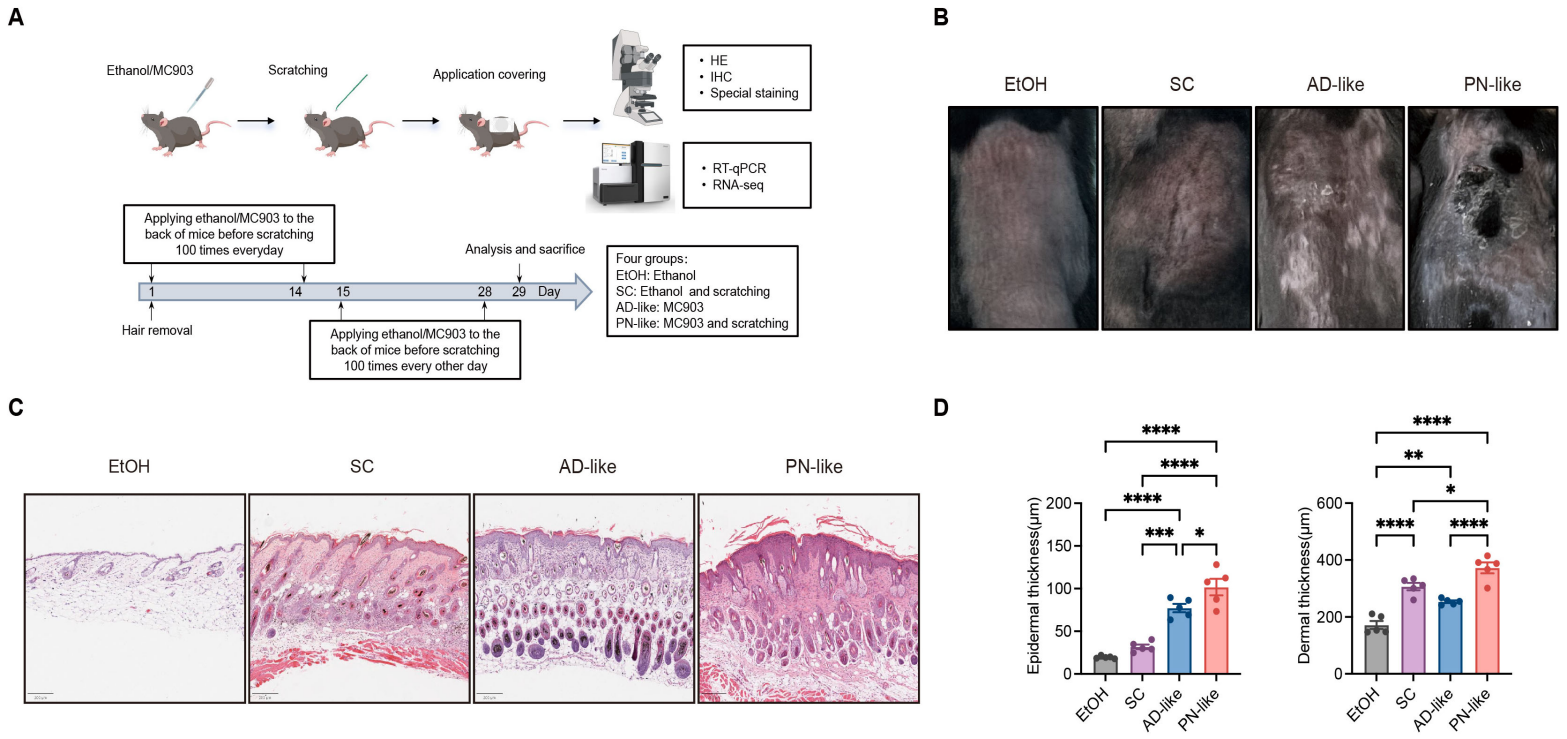
Phenotypic characteristics of PN-like mouse models. **(A)** Schematic diagram illustrating the modeling process for the four experimental groups. N = 5 mice per group. **(B)** Representative gross appearance of the back lesions of four murine groups. **(C)** On day 29, skin tissues were collected from all groups. Representative H&E-stained images of murine skin tissues are shown. Scale bar: 200 μm. **(D)** Epidermal and dermal thickness were measured from scanned H&E images using QuPath software (v0.5.1). Data are presented as mean ± SEM. EtOH, ethanol; SC, scratching; AD, atopic dermatitis; PN, prurigo nodularis; H&E, hematoxylin and eosin. *P < 0.05, **P < 0.01, ***P < 0.001, ****P < 0.0001.

We successfully established a PN-like murine model through the combined application of topical MC903 and mechanical scratching stimulation (**Figure 1A**). As shown in **Figure 1B**, both the MC903-induced AD-like model and the PN-like model exhibited gross signs of inflammation, with the PN-like model showing more prominent raised plaques. However, nodular lesions were not evident in the PN-like model, necessitating histological analysis for further characterization. Histological examination revealed that the skin lesions in PN-like mice partially mirrored those observed in PN patients (27), including hyperkeratosis, pseudoepitheliomatous hyperplasia with hypergranulosis, thickened collagen, dilated and thinned blood vessels in the superficial dermis (**Figure 1C**). Among the four experimental groups, the PN-like mice exhibited the most significant increase in both epidermal and dermal thickness. Compared to the AD-like group, the SC group mice showed thicker dermal layers but thinner epidermal layers (**Figure 1D**).

### 3.2 Aberrant keratin expression and excessive collagen deposition in PN-like mice

**Figure 2:**
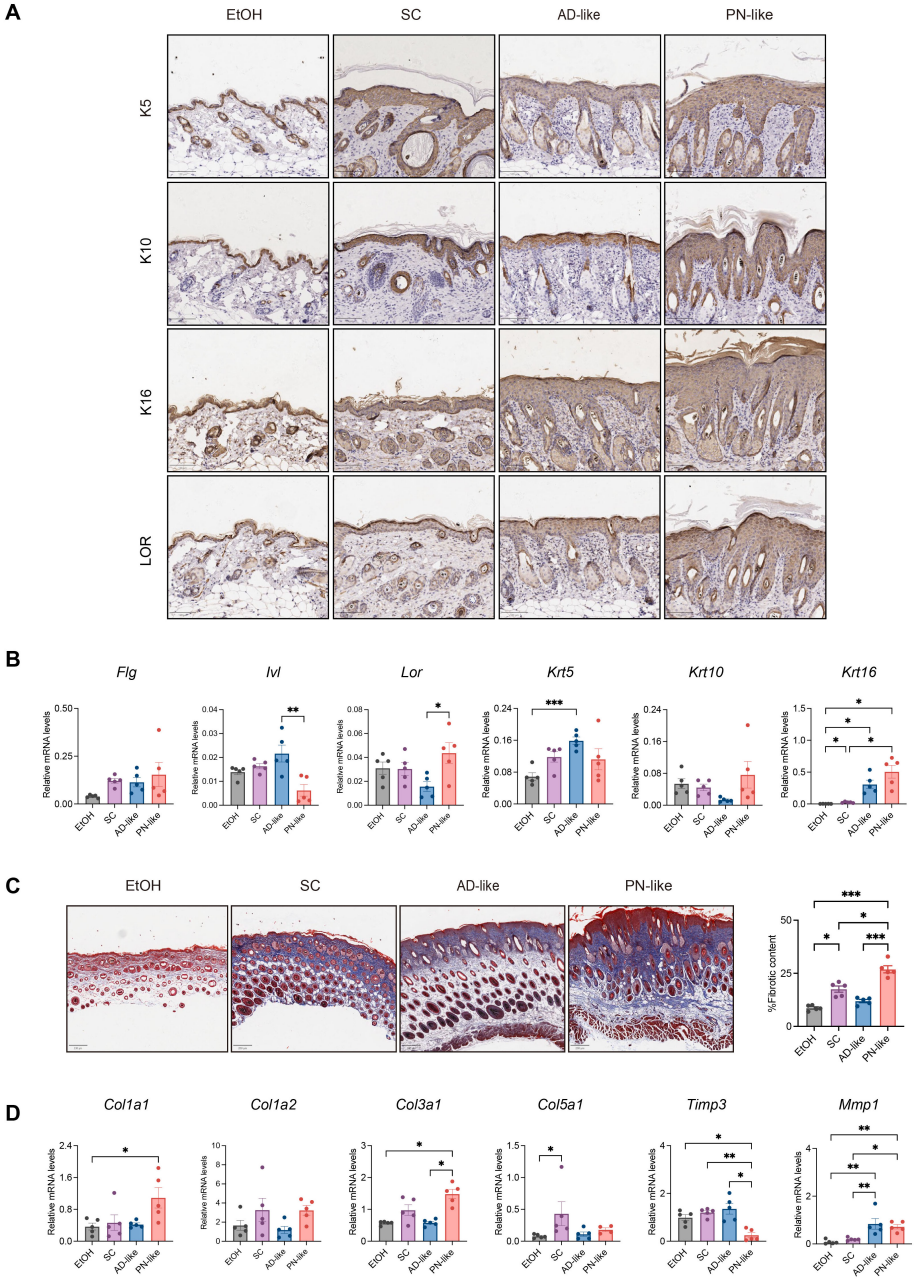
Dysregulated keratin expression and prominent collagen accumulation in PN-like lesions. **(A)** Representative IHC images of keratin markers (K5, K10, K16) and LOR across experimental groups; scale bar: 100 μm. **(B)** Quantitative RT-PCR analysis of epidermal differentiation-related genes. **(C)** Representative MT images for collagen fibers (left panel) and statistical analysis of collagen fibers-positive areas (right panel); scale bar: 200 μm; **(D)** Quantitative RT-PCR analysis of collagen-related genes. Data are presented as mean ± SEM. EtOH, ethanol; SC, scratching; AD, atopic dermatitis; PN, prurigo nodularis; MT, Masson’s trichrome; IHC, immunohistochemistry. *P < 0.05, **P < 0.01, ***P < 0.001.

Abnormal keratins expression was present in PN lesional skin (28). To investigate whether the expression pattern of keratins in PN-like mouse skin recapitulates that of PN patients, IHC was conducted to assess the distribution of key keratin markers, including keratin 5 (K5), keratin 10 (K10), keratin 16 (K16), and loricrin (LOR) in the skin lesions of four murine groups. In all groups, K5 and K16 were predominantly localized in the basal layer and lower suprabasal layers, while K10 and LOR were mainly expressed in the granular layer and upper suprabasal layers (**Figure 2A**). The expression levels of *Lor* and *Krt16* were significantly upregulated, while *Ivl* expression was markedly downregulated in the PN-like mouse models (**Figure 2B**).

Dermal fibrosis is a well-known histological feature of PN (29). MT staining revealed significant collagen fiber proliferation in PN-like mice compared to the other three groups (**Figure 2C**). The expression of fibrosis-related genes, including *Col1a1*, *Col3a1* and *Mmp1* was markedly upregulated, while *Timp3* expression was downregulated in PN-like mice (**Figure 2D**).

### 3.3 Hyperactive inflammatory responses in PN-like mice

**Figure 3:**
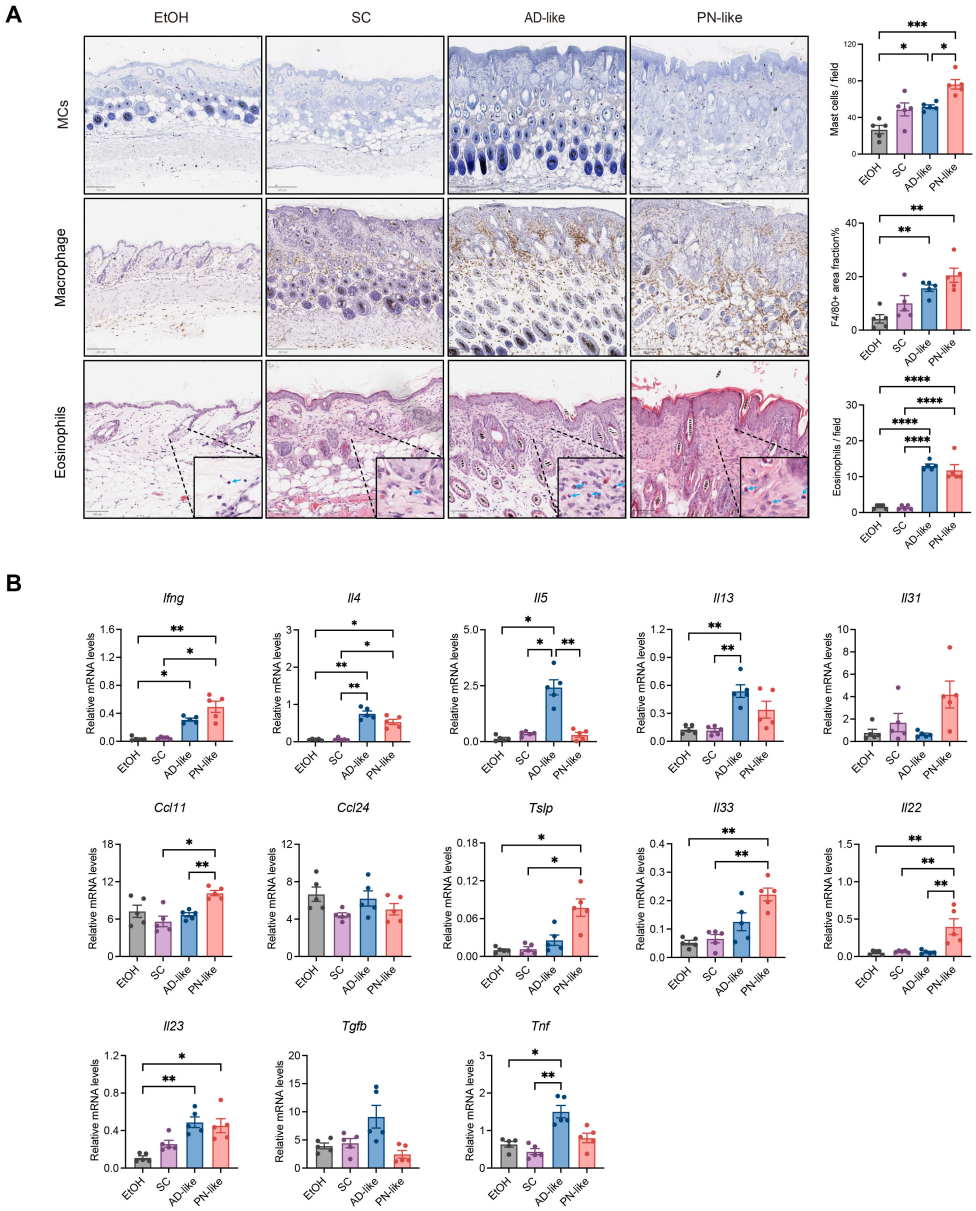
PN-like mice show significantly enhanced inflammatory activity. **(A)** Left panel: representative IHC images of MCs, macrophages and eosinophils. Right panel: statistical analysis of positively stained cells. Scale bar: 200 μm (MCs and macrophages) or 100 μm (eosinophils); **(B)** mRNA expression levels of inflammatory cytokines measured by quantitative RT-PCR. Data are presented as mean ± SEM. EtOH, ethanol; SC, scratching; AD, atopic dermatitis; PN, prurigo nodularis; MC, mast cells. *P < 0.05, **P < 0.01, ***P < 0.001.

The inflammatory response, characterized by immune cell infiltration and the release of inflammatory mediators, plays a central role in the pathogenesis of PN (10). Our investigation focused on the density and distribution patterns of MCs, macrophages, and eosinophils in skin tissues. As demonstrated in **Figure 3A**, MCs in AD-like mice were predominantly localized in the papillary dermis, whereas in SC mice, MCs were mainly distributed in the deep dermis and subcutaneous layer. Notably, PN-like mice exhibited a diffuse distribution of MCs throughout the entire dermis and subcutaneous layer with significantly increased MC numbers, accompanied by similarly elevated macrophage and eosinophil counts in both AD-like and PN-like mice compared to controls. The distribution pattern of macrophages in mouse skin closely resembled that of mast cells. The number of macrophages and eosinophils was notably increased in both AD-like and PN-like mice.

Additionally, we analyzed the expression levels of inflammatory cytokines. Cytokine analysis demonstrated that PN-like lesions showed upregulated expression of Th1-related (*Ifng*), Th17-related gene (*Il23*), and Th17/Th22-related gene (*Il22*) genes. The characteristic Th2-related cytokines (*Il4*, *Il5*, *Il13*) were markedly increased in AD-like lesions, while *Il4* and *Ccl11* expression was elevated to various extent in PN-like lesions (**Figure 3B**) appeared to be associated with dermal eosinophilia. The epithelial cell–derived cytokines *Tslp* and *Il33* were significantly elevated in PN-like lesions (**Figure 3B**).

### 3.4 IENFD and blood vessels in PN-like mice

We assessed the distribution of nerve fibers in the skin of PN-like mice using the IENFD method. The results showed that PN-like mice exhibited the highest IENFD, followed by AD-like mice, with both PN-like and AD-like mice showing prominent nerve fiber branching in the epidermis (**Supplementary Figure S1A**). This finding contrasts with the reduced IENFD observed in the skin lesions of PN patients (30), which may be attributed to differences in the timing and extent of skin structural damage. Additionally, we examined the number and morphology of blood vessels in the superficial dermis. Additionally, we quantified vascular density in the superficial dermis. We detected the vascular endothelial marker CD31 in lesions of PN-like mice by IHC and found that PN-like mice exhibited the highest vascular density among all experimental groups (**Supplementary Figure S1B**).

### 3.5 PN-like mice demonstrate a distinct pattern of transcriptome

**Figure 4:**
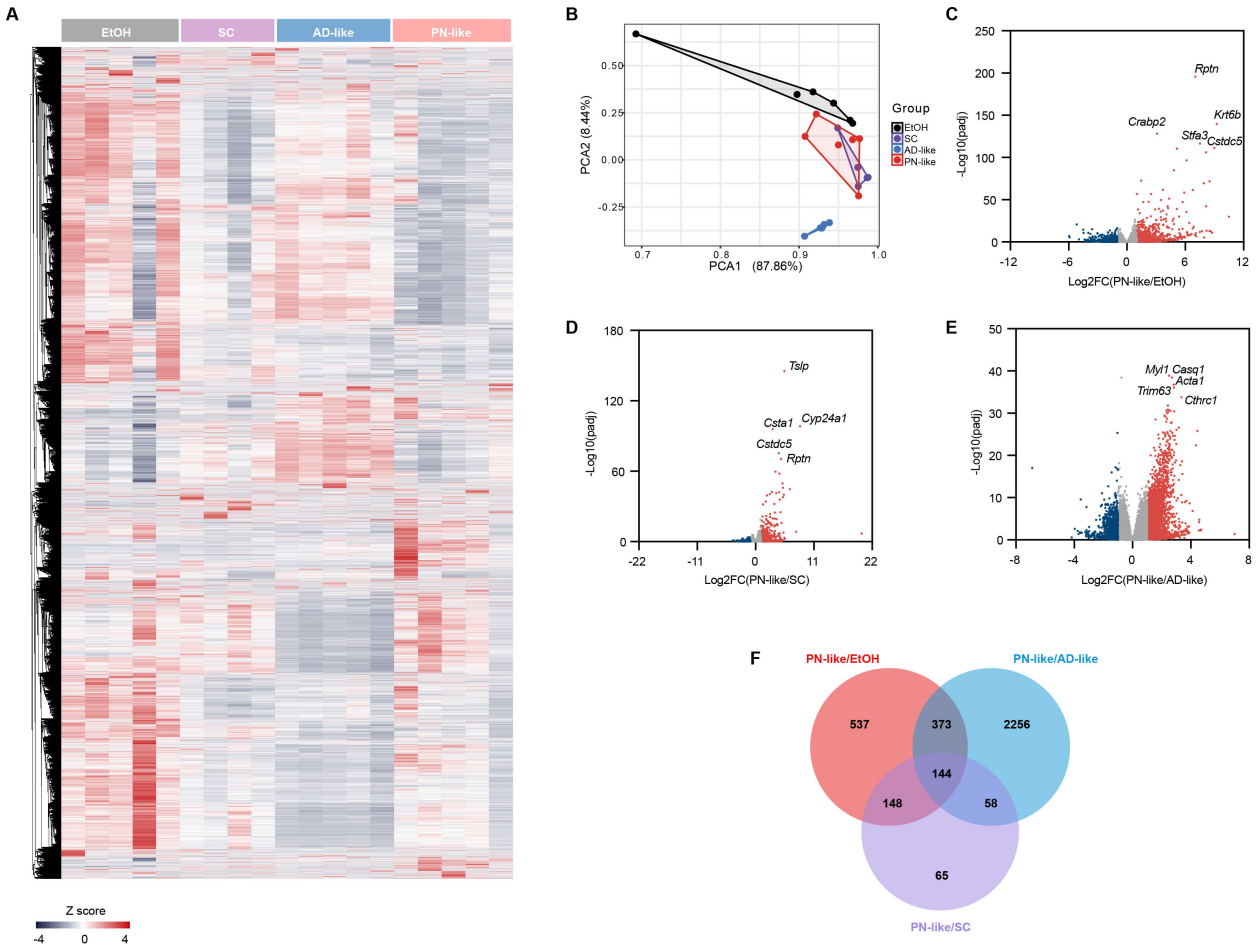
Distinct transcriptomic profile of the PN-like murine model. **(A)**Heatmap of all genes analyzed by RNA-seq in the skin of groups of EtOH(n=5), SC(n=4), AD-like (n=5), and PN-like (n=5) groups. The color scale represents the row z scores. **(B)** PCA of skin transcriptome profiles in the four murine models. Each point represents an individual sample. (**c-e**) Volcano plot comparing gene expression between **(C)** PN-like and EtOH skin, **(D)** PN-like and SC groups, and **(E)** PN-like and AD-like groups. Red points denote significantly upregulated DEGs (log2 FC > 1, FDR < 0.05), while blue points represent significantly downregulated DEGs (log2 FC < -1, FDR < 0.05). **(F)** Venn diagram showing the overlap of DEGs among the PN-like, EtOH, SC, and AD-like groups. EtOH, ethanol; SC, scratching; AD, atopic dermatitis; PN, prurigo nodularis; PCA, principal component analysis; DEG, differentially expressed genes; FC, fold change; FDR, false discovery rate.

In **Figure 4A**, skin transcriptome profiles were performed in four murine models: the ethanol EtOH group (n = 5), the SC group (n = 4), the AD-like group (n = 5), and the PN-like group (n = 5). Principal component analysis effectively distinguished the PN-like group from the EtOH and AD-like groups, but not from the SC group (**Figure 4B**), indicating a similar transcriptional pattern between the PN-like and SC groups with a |log2 fold change| > 1 and FDR < 0.05 (**Figures 4C-E**). We performed an intersection analysis of DEGs between the PN-like group and the other three groups of mice and identified 144 DEGs that were unique to the PN-like group (**Figure 4F**). The full lists of DEGs for all comparisons were provided in **Supplementary Table S3**.

### 3.6 The functional enrichment of DEGs in the PN-like model

**Figure 5:**
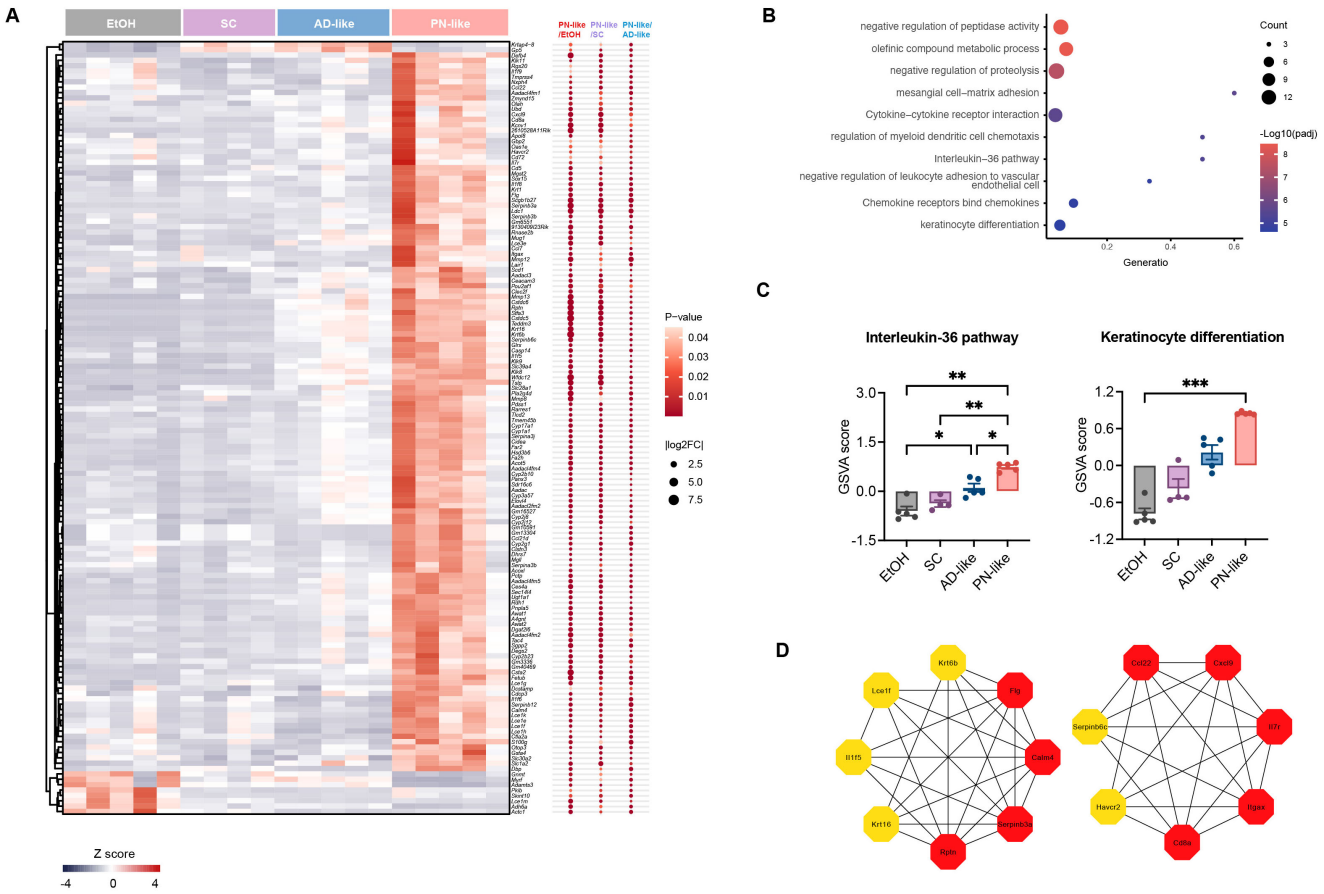
Functional enrichment analysis of DEGs in the PN-like murine model. **(A)** Heatmap displaying the expression patterns of 144 overlapping DEGs across three comparison groups: PN-like *vs*. EtOH, PN-like *vs*. SC, and PN-like *vs*. AD-like. The color scale represents row z-scores, indicating standardized gene expression levels. **(B)** Functional enrichment pathways of the 144 DEGs. **(C)** GSVA results illustrating the activity scores of the IL-36 signaling pathway and keratinocyte differentiation pathway across the four murine models. **(D)** The top 15 genes ranked by the MCC algorithm among the 144 DEGs were identified as hub genes. Data are presented as mean ± SEM. EtOH, ethanol; SC, scratching; AD, atopic dermatitis; PN, prurigo nodularis; DEG, differentially expressed genes; FC, fold change; GSVA, gene set variation analysis; IL, interleukin; MCC, maximal clique centrality. *P < 0.05, **P < 0.01, ***P < 0.001.

Based on the functional annotations in Gene Ontology, Kyoto Encyclopedia of Genes and Genomes and Reactome Gene Sets, we further analyzed the biological function of 144 specific DEGs in the PN-like model (**Figure 5A**). Our analysis revealed that DEGs were mainly enriched in the pathways related to protein metabolism, cytokine-chemokine interactions, regulation of myeloid dendritic cell chemotaxis, IL-36 pathway, negative regulation of leukocyte adhesion to vascular endothelial cell, and keratinocyte differentiation (**Figure 5B**). Notably, previous studies have reported significant upregulation of IL-36 family genes in lesions of PN patients (31). To further explore this observation, GSVA of the IL-36 pathway across the four groups revealed marked upregulation in PN-like mice and moderate upregulation in AD-like mice relative to the EtOH group, while PN-like mice also exhibited substantial upregulation of the keratinocyte differentiation pathway (**Figure 5C**). To further identify hub genes among the 144 DEGs specifically expressed in the PN-like murine model, we combined PPI network analysis with the MCC algorithm from the CytoHubba plugin and identified 15 hub genes: *Itgax*, *Calm4*, *Cd8a*, *Ccl22*, *Rptn*, *Il7r*, *Cxcl9*, *Flg*, *Serpinb3a*, *Il1f5*, *Krt16*, *Krt6b*, *Serpinb6c*, *Havcr2* and *Lce1f* (**Figure 5D**).

A notable proportion of PN patients have a history of atopy or current atopic comorbidity (12), such as AD. This clinical observation aligns with our PN-like mouse model, which was established on an AD-like background. To delineate the molecular distinctions between PN-like and AD-like, we analyzed the 2256 unique DEGs identified from the PN-like *vs*. AD-like comparison (**Figure 4F**) through functional enrichment analysis. These DEGs were predominantly enriched in pathways related to keratinization, inflammatory response, cell migration, extracellular matrix organization (ECM), and blood vessel development (**Supplementary Figure S2A**). PN-like mice and SC mice showed higher GSVA scores of ECM compared to AD-like mice (**Supplementary Figure S2B**).

### 3.7 Robust transcriptional signaling of Th17/Th22 skewing and fibrotic responses in PN-like mice

**Figure 6:**
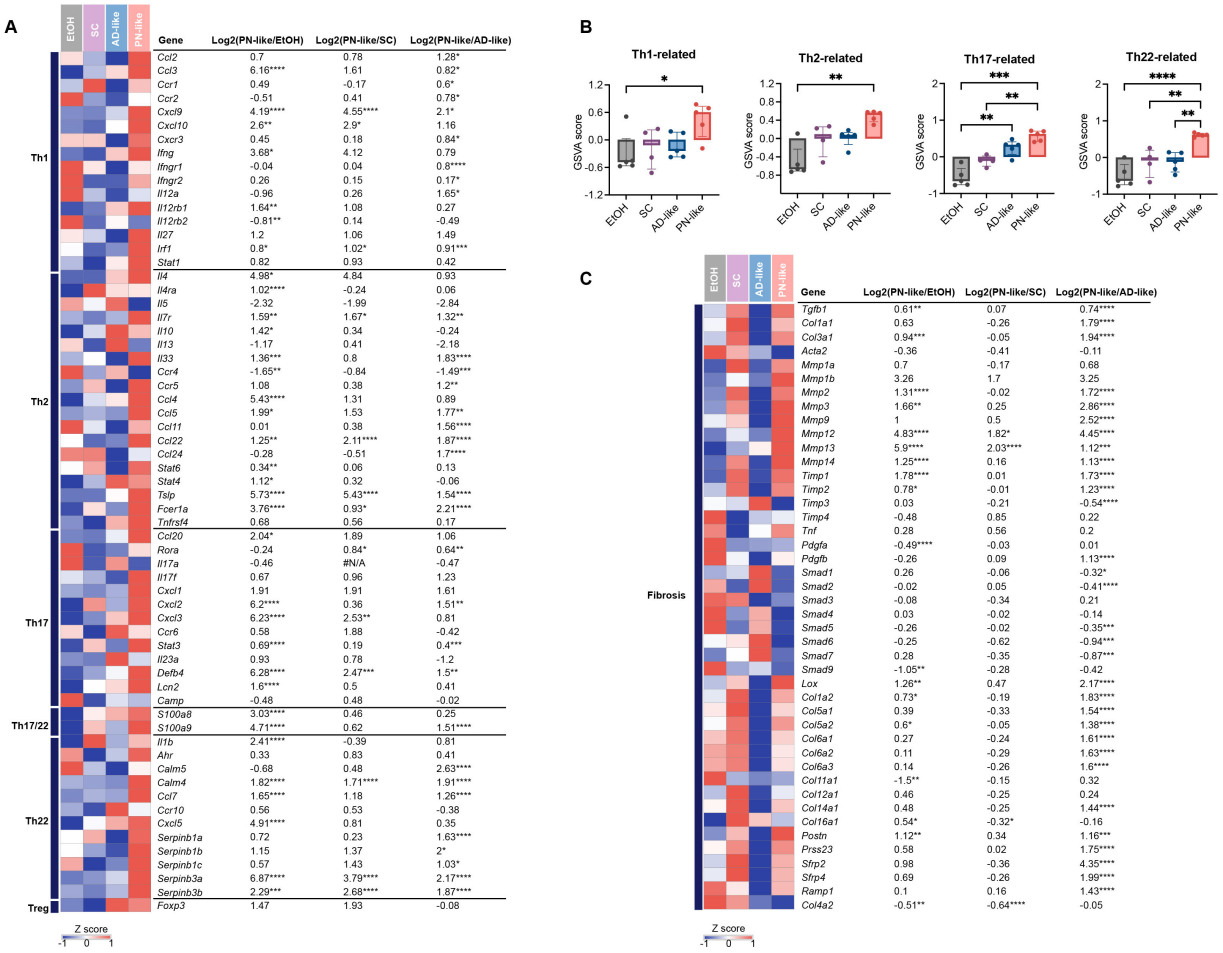
Distinct immune and fibrotic signatures in the PN-like murine model revealed by cutaneous gene expression profiling. **(A)** Heatmap depicting the average gene expression of T cell subsets across the four murine models. The color code represents the row z scores. **(B)** GSVA score comparison of T cell subset-related genes (Th1, Th2, Th17, and Th22) in the skin of the four murine models. **(C)** Heatmap illustrating the average expression of fibrosis-related genes across the four murine models. Data are presented as median ± interquartile range. EtOH, ethanol; SC, scratching; AD, atopic dermatitis; PN, prurigo nodularis; GSVA, gene set variation analysis; Th, T helper. *P < 0.05, **P < 0.01, ***P < 0.001, ****P < 0.0001.

To characterize the immune phenotypes in PN-like mice and their resemblance to human PN lesions, we analyzed the transcriptional expression of marker genes associated with distinct CD4^+^ T cell subsets (32). RNA-seq revealed significant upregulation of key immune markers in PN-like mice compared to the other three groups, including *Cxcl9* (Th1-associated gene), *Ccl22* and *Tslp* (Th2-associated genes), *Defb4* (Th17-associated gene), and *Calm4*, *Serpinb3a*/*3b* (Th22-associated genes). Furthermore, PN-like mice exhibited pronounced increases in Th17/IL17-associated genes (*Ccl20*, *Cxcl2*/*3*, *Lcn2*), Th17/Th22-associated genes (*S100a8/a9*) and Th22/IL22-associated genes (*Il1b*, *Calm5*, *Ccl7*, *Cxcl5*, *Sepinb1a/1b/1c*) compared to the EtOH and/or AD-like groups (**Figure 6A**). To quantify these observations, GSVA was performed to compare the expression of Th1, Th2, Th17, and Th22 gene markers. The analysis demonstrated robust upregulation of Th22-related genes in PN-like mice relative to all other groups, significant elevation of Th17 markers compared to the EtOH and AD-like groups, and modest increases in Th1 and Th2 markers compared to the EtOH group (**Figure 6B**). These findings collectively indicate that the immune phenotype in PN-like mice closely recapitulates the Th17/Th22-skewed immune signature observed in human PN lesions.

Previous studies have demonstrated a distinct mesenchymal dysregulation in the dermal fibrosis of PN, which is marked by enhanced angiogenesis, fibrosis, and extracellular matrix production (29). In PN-like mice, there was a notable upregulation of collagen-encoding genes (*Col1a1/a2, Col3a1*, etc.) and matrix metalloproteinase-associated genes (*Mmp2*/*3*/*9*/*12*, etc.) to varying extent. Additionally, recently identified fibroblast marker genes in PN skin (33, 34), including *Postn*, *Prss23*, *Srfp2/4*, *Ramp*, showed a significant increase in PN-like mice (**Figure 6C**). Furthermore, genes related to vessel function, such as *Angpt1/2*, *Vcam1*, *Nos3* and *Il6*, were enhanced in PN-like mice (**Supplementary Figure S3**).

Moreover, the mRNA expression of skin barrier proteins, including keratins (*Krt1/10/16*), filaggrin *(Flg)*, loricrin *(Lor)*, kallikreins (*Klk9/11*), as well as neural remodeling proteins like nerve growth factor (*Ngf)* and substance P (*Tac1*), was elevated to varying extents in PN mice (**Supplementary Figure S3**).

To further evaluate the similarity between the PN-like murine model and PN patients, we compared the 144 DEGs with those from human PN skin lesions (32). The results demonstrate that while the PN-like mouse model effectively recapitulates the upregulated gene expression profile observed in human PN, its capacity to replicate downregulated gene signatures remains limited (**Supplementary Table S4**).

## 4 Discussion

The lack of representative animal models and the limited efficacy of current targeted therapies in nodular lesions underscore the urgent need for reliable experimental models of PN (8, 12–14). In this study, we established a PN-like mouse model driven by type 2 inflammation and mechanical scratching that recapitulates key pathological features observed in PN patients, including aberrant epidermal proliferation and differentiation, pronounced dermal fibrosis, mixed T helper cell - driven immune activation (Th1/Th2/Th17/Th22), and heightened cutaneous inflammatory responses.

Notably, the PN-like lesions in our model exhibited significant thickening of the epidermis, consistent with observations in human PN nodules. IHC and transcription analysis further revealed aberrant expression of markers involved in keratinocyte proliferation and differentiation, such as K16, LOR and IVL, indicating disrupted keratinocyte homeostasis. Keratins play critical roles in epidermal structural integrity and barrier homeostasis, as well as in wound repair and inflammation (35–37). In particular, K16 is a well-established stress-responsive keratin that function as an early alarmin in damaged skin (38), whose expression is strongly induced by scratching-induced keratinocyte stress and mechanical damage to subsequently modulate keratinocyte proliferation and inflammatory responses (31, 39). Epidermal K16 expression was upregulated at both the transcriptional and translational levels in our PN-like mouse model, which is consistent with the enhanced K16 expression in the lesional skin of PN patients (28). The skin barrier protein LOR showed granular layer localization in both AD-like and PN-like mouse models, consistent with its expression pattern in human AD and PN lesions. While lesional skin from AD and PN patients exhibited increased LOR and involucrin (IVL) but decreased filaggrin (FLG) at the protein level (40), our results revealed elevated *Lor* but reduced *Ivl* expression at the transcription level in PN-like models compared to AD-like mice, with unchanged *Flg* levels. This differential expression pattern suggests compensatory mechanisms among barrier proteins that ultimately fail to restore skin barrier homeostasis under pathological conditions (40).

Dermal fibrosis is a well-established histopathological hallmark of PN (29). Transcriptomic data of PN patients have consistently demonstrated profound dysregulation in the interstitial compartment compared to AD, characterized by fibroblast activation, excessive ECM deposition, and fibrotic remodeling, as evidenced by prior studies (33, 41, 42). In our PN-like murine model, both H&E and MT staining confirmed excessive collagen accumulation within the superficial dermis, recapitulating the fibrotic features observed in PN lesions. Transcriptional analysis in a PN-like mouse model revealed upregulation of collagen-encoding genes (e.g., *Col1a1*, *Col3a1*), while ECM regulatory genes exhibited opposing trends - specifically, *Mmp1* expression was elevated, and its negative regulator *Timp3* was downregulated. This paradoxical expression pattern, where increased *Mmp1* and decreased *Timp3* coincide with heightened collagen deposition, contrasts with the expected ECM degradation phenotype. Notably, this dysregulation paralleled the expression profile in PN patients, showing elevated *MMP1* and reduced *TIMP3* expression (32), yet the mechanism remains poorly understood. Intriguingly, in AD lesions, *MMP1* expression is also elevated in both the epidermis and dermis (43), and serum MMP-1 levels correlate with the severity of epidermal barrier dysfunction (44–46). The upregulation of *Mmp1* in both AD-like and PN-like mouse models suggests that *MMP1* could be involved not only in collagen remodeling but also in epidermal dysfunction. These findings demonstrate that the dermal fibrosis in PN-like mice closely mirrors the structural and molecular alterations seen in PN nodular lesions, validating the model’s relevance for mechanistic and therapeutic studies.

Mixed infiltration of immune cells and subsequent robust inflammatory responses in the dermis are central to the pathogenesis of PN (7). PN lesions demonstrated significantly enhanced type 2 inflammatory responses relative to healthy controls, their intensity remained attenuated compared to AD (29). Our study revealed that canonical Th2 cytokines (*Il4*, *Il5*, *Il13*) demonstrate the highest expression levels in AD-like mice consistent with findings from 14-day AD-like models (47), and followed by PN-like models, while type 2-associated immune cells displayed differential infiltration patterns - macrophages reached peak levels in PN-like mice, with eosinophil accumulation second only to AD-like specimens. GSVA suggested PN-like mice exhibit the most prominent Th2 signatures. PN-like mice exhibited the highest transcriptional expression of *Ifng* and the greatest Th1 response GSVA score, aligning with the Th1-dominant immune phenotype seen in PN patient lesional skin - likely driven by scratching-induced keratinocyte stress responses (29). Notably, the characteristic enrichment of IL-17, IL-22 and IL-36 in PN (versus AD) may underlie its clinical overlap with psoriasis (31, 32), a mechanism validated in our PN-like model through GSVA demonstrating robust Th17/Th22 responses and IL-36 pathway activation in lesional skin.

In PN patients, lesions showed decreased IENFD but increased nerve fiber branching in the epidermis, likely due to chronic scratching-induced fragmentation and subsequent sprouting of nerve fibers (39). Interestingly, our PN-like models exhibited increased IENFD, suggesting this structural preservation may relate to differences in scratching intensity/duration or different species. The study by Yamaoka et al. describes a transient increase in IENFD following a single episode of skin scratching, which resolves within 2–3 weeks (48). In contrast, our model employs repeated, chronic scratching for 28 days. We find that the inflammatory background appears to be a key driver of this sustained neuroproliferation as evidenced by enhanced nerve fiber branching in both AD-like and PN-like models. We hypothesize that chronic inflammation, in synergy with mechanical injury, drives a persistent and potent “sprouting phase”, which appears to be supported by the elevated expression of the branching-associated gene *NGF* in both PN patients (39) and our PN-like models. Intriguingly, the Hashimoto prurigo-like model showed more pronounced nerve fiber sprouting and NGF expression at peripheral lesions versus central lesions (49). Unfortunately, we didn’t compare lesions with adjacent non-lesional skin. The alterations in IENFD and branching patterns are likely independent, and only through two- or three-dimensional assessment of both parameters can a comprehensive neuroanatomical profile be established (30). Our preliminary investigations have identified dermal nerve fiber bundles in PN-like murine models that demonstrate morphological similarities to PN patient lesions, although the current lack of standardized quantification protocols for dermal nerve fibers precludes definitive comparative analysis.

CD31 IHC revealed a significant increase in superficial dermal vascular density in the PN-like group compared to the EtOH group, consistent with the characteristic vascular hyperplasia observed in lesional skin of PN patients (50). Notably, epidermal thickness showed strong correlation with vascular density (50), and our data demonstrated that PN-like mice exhibited both the thickest epidermis and highest vascular counts among all groups. Furthermore, GSVA identified significant enrichment of vessel development-related gene signatures in PN-like mice. Collectively, these findings indicate robust neovascularization in the superficial dermis of PN-like murine lesions.

In summary, the PN-like mouse model we established under a type 2 inflammatory background effectively recapitulates the nodular skin lesions of PN patients in terms of histological alterations, inflammatory cell infiltration, and immune cell composition. However, several limitations should be acknowledged: First, the current method failed to recapitulate characteristic nodular lesions. Second, the model inadequately addresses disease comorbidities, focusing solely on type 2 inflammation background. Given that PN patients frequently present with type 2 diabetes, chronic kidney disease, etc (51), it is dispensable to elucidate pathogenic mechanisms across different comorbidity backgrounds. Third, our PN-like model shows deficiencies in recapitulating neural remodeling including species-divergent IENFD changes. Our future investigations will implement the following strategic modifications: (1) extending the duration of mechanical stimulation to simulate long-term consequences of chronic scratching; (2) incorporating pruritogen-induced spontaneous scratching behavior to establish a more authentic, stimulus-independent itch-scratch cycle; and (3) combining exogenous administration of key cytokines (e.g., IL-31) to synergistically drive both pruritic and pro-fibrotic pathways.

## Data Availability

The original contributions presented in the study are publicly available. The data have been deposited in the Mendeley Data repository under accession number [10.17632/my59z5zsvh.1](https://data.mendeley.com/datasets/my59z5zsvh/1).

## Supplementary material

Supplementary Figure 1 Increased IENFD and blood vessels in PN-like mice. **(A)** Representative IHC images for PGP9.5 (left panel) and statistical analysis of IENFD (right panel) in the four experimental groups; scale bar: 20 μm. **(B)** Representative IHC images for CD31 (left panel) and statistical analysis of CD31-positive areas (right panel) in the four experimental groups; scale bar: 100 μm. Data are presented as mean ± SEM. EtOH, ethanol; SC, scratching; AD, atopic dermatitis; PN, prurigo nodularis; IHC, immunohistochemistry. **P* < 0.05, ***P* < 0.01, ****P* < 0.001.

Supplementary Figure 2 Functional enrichment analysis of DEGs in PN-like versus AD-like murine models. **(A)** Functional enrichment pathways of the 2,256 unique DEGs identified from the PN-like *vs*. AD-like comparison. **(B)** Comparison of GSVA scores for the 2,256 unique DEGs across the four murine models. Data are presented as mean ± SEM. EtOH, ethanol; SC, scratching; AD, atopic dermatitis; PN, prurigo nodularis; DEG, differentially expressed genes; GSVA, gene set variation analysis; ECM, extracellular matrix organization. **P* < 0.05, ***P* < 0.01, ****P* < 0.001, *****P* < 0.0001.

Supplementary Figure 3 Differential expression of vessel function, skin barrier, and neural remodeling-related genes in PN-like mice. Heatmap illustrating the average expression of genes associated with vessel function, skin barrier and neural remodeling across the four murine models. The color code represents the row z scores. EtOH, ethanol; SC, scratching; AD, atopic dermatitis; PN, prurigo nodularis. **P* < 0.05, ***P* < 0.01, ****P* < 0.001, *****P* < 0.0001.

Supplementary Table 1 RT-qPCR primers. EtOH, ethanol; SC, scratching; AD, atopic dermatitis; PN, prurigo nodularis.

Supplementary Table 2 Gene sets for GSVA. EtOH, ethanol; SC, scratching; AD, atopic dermatitis; PN, prurigo nodularis.

Supplementary Table 3 DEGs list of PN-like versus the other three groups. EtOH, ethanol; SC, scratching; AD, atopic dermatitis; PN, prurigo nodularis.

Supplementary Table 4 The overlapping genes between the PN-like murine model and human PN. The data in the table are displayed as log2FC along with adjusted p-values. EtOH, ethanol; SC, scratching; AD, atopic dermatitis; PN, prurigo nodularis; FC, fold change. **P* < 0.05, ***P* < 0.01, ****P* < 0.001, *****P* < 0.0001.
