# Supplementary figures and images for: A murine model of prurigo nodularis-like skin lesions induced by persistent scratching under type 2 inflammatory conditions
Source: Front Immunol. 2025 Oct 28;16:1648830. doi: 10.3389/fimmu.2025.1648830 (PMC12602216; doi:10.3389/fimmu.2025.1648830)

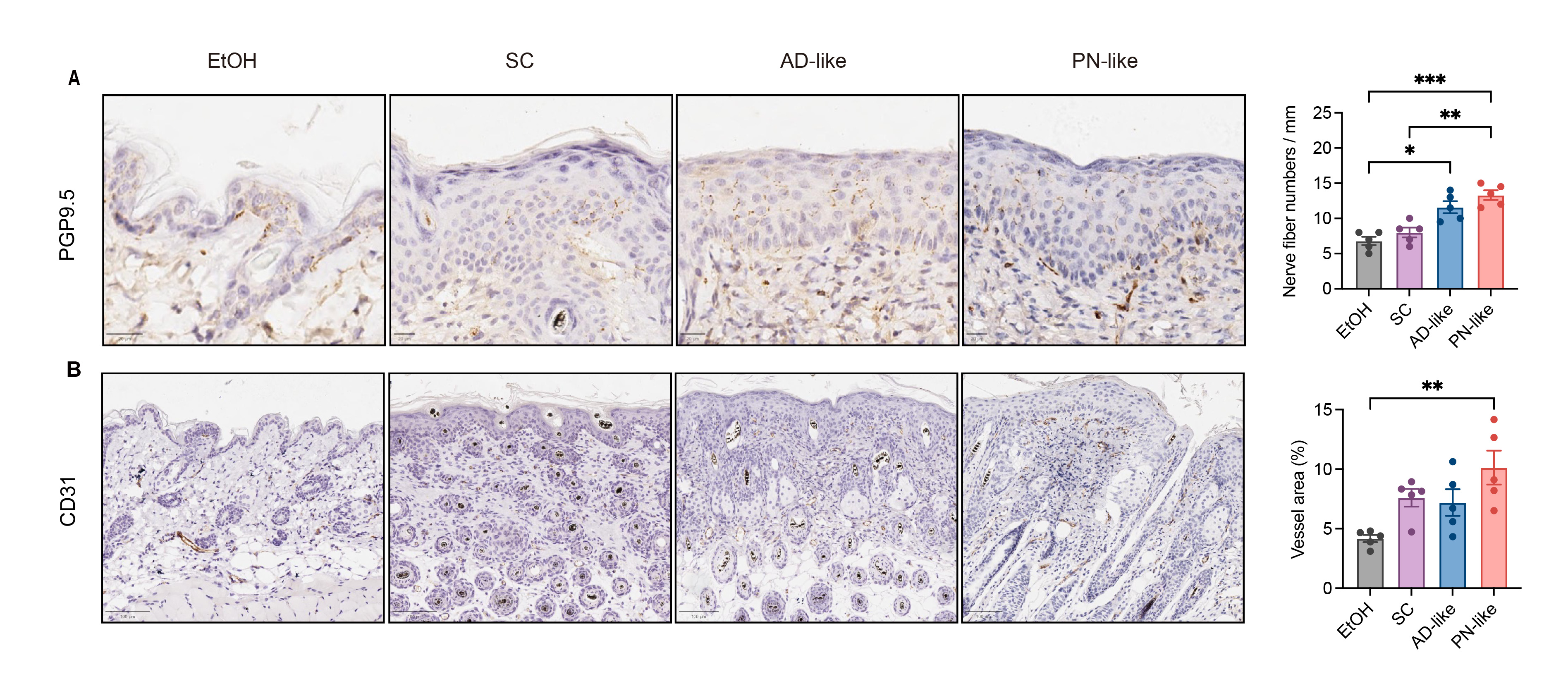

Supplement: Supplementary Figure 1 — Increased IENFD and blood vessels in PN-like mice. (A) Representative IHC images for PGP9.5 (left panel) and statistical analysis of IENFD (right panel) in the four experimental groups; scale bar: 20 μm. (B) Representative IHC images for CD31 (left panel) and statistical analysis of CD31-positive areas (right panel) in the four experimental groups; scale bar: 100 μm. Data are presented as mean ± SEM. EtOH, ethanol; SC, scratching; AD, atopic dermatitis; PN, prurigo nodularis; IHC, immunohistochemistry. *P < 0.05, **P < 0.01, ***P < 0.001. [file Image1.jpeg]

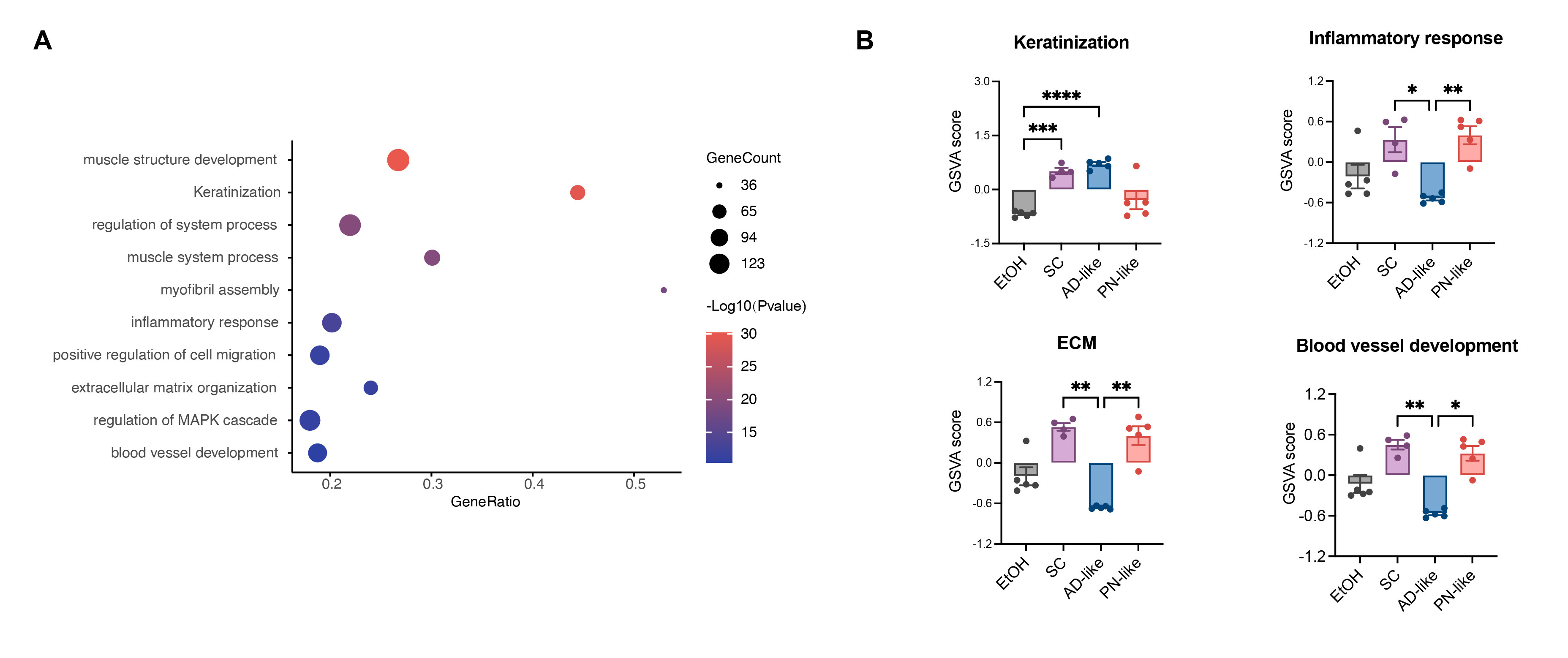

Supplement: Supplementary Figure 2 — Functional enrichment analysis of DEGs in PN-like versus AD-like murine models. (A) Functional enrichment pathways of the 2,256 unique DEGs identified from the PN-like vs. AD-like comparison. (B) Comparison of GSVA scores for the 2,256 unique DEGs across the four murine models. Data are presented as mean ± SEM. EtOH, ethanol; SC, scratching; AD, atopic dermatitis; PN, prurigo nodularis; DEG, differentially expressed genes; GSVA, gene set variation analysis; ECM, extracellular matrix organization. *P < 0.05, **P < 0.01, ***P < 0.001, ****P < 0.0001. [file Image2.jpeg]

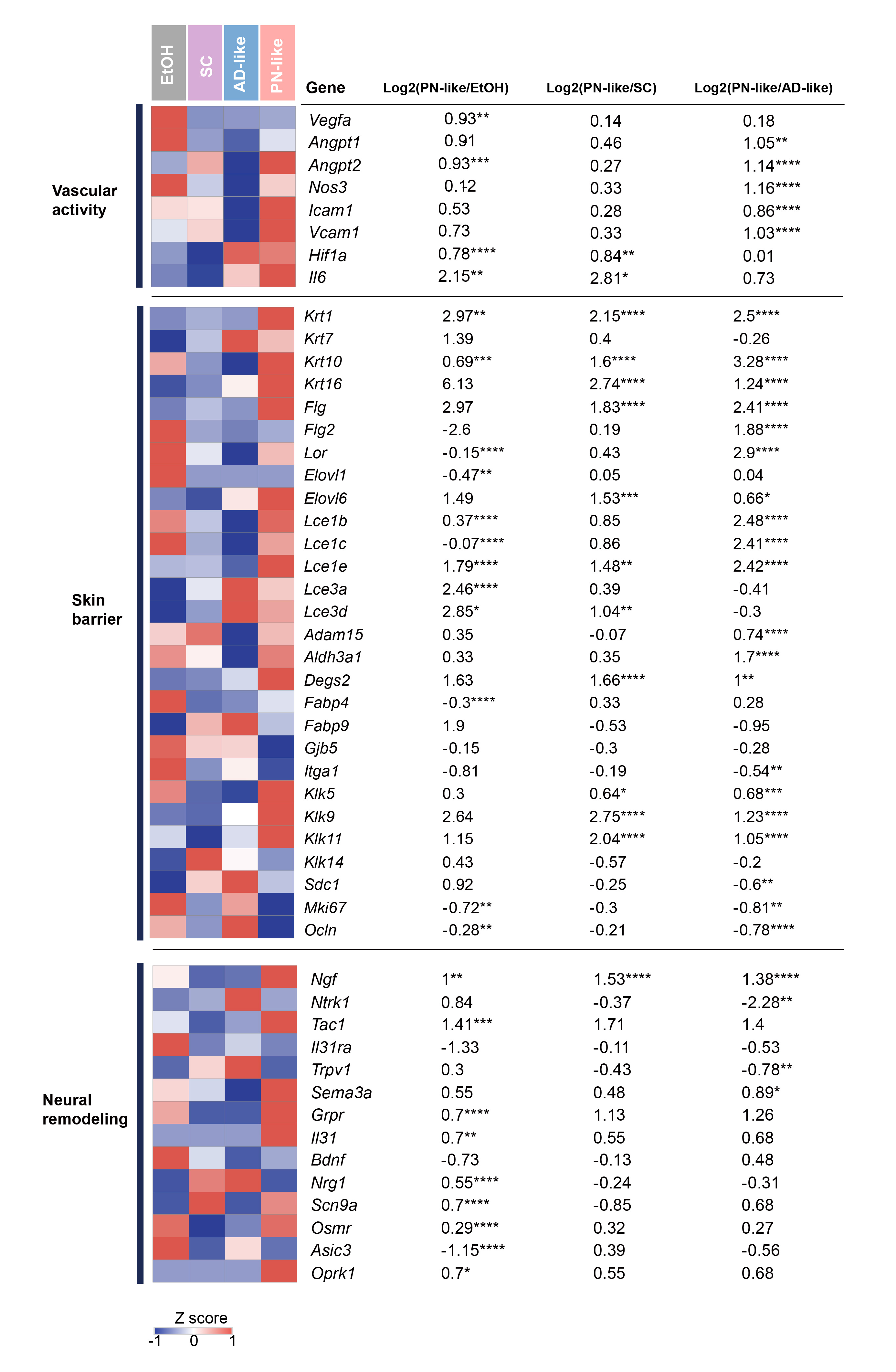

Supplement: Supplementary Figure 3 — Differential expression of vessel function, skin barrier, and neural remodeling-related genes in PN-like mice. Heatmap illustrating the average expression of genes associated with vessel function, skin barrier and neural remodeling across the four murine models. The color code represents the row z scores. EtOH, ethanol; SC, scratching; AD, atopic dermatitis; PN, prurigo nodularis. *P < 0.05, **P < 0.01, ***P < 0.001, ****P < 0.0001. [file Image3.jpeg]
